# Interaction effects of sex on the sleep loss and social jetlag-related negative mood in Japanese children and adolescents: a cross-sectional study

**DOI:** 10.1093/sleepadvances/zpac035

**Published:** 2022-09-21

**Authors:** Takae Shinto, Yu Tahara, Aato Watabe, Naomichi Makino, Masataka Tomonaga, Haruo Kimura, Yuki Nozawa, Kazuki Kobayashi, Masaki Takahashi, Shigenobu Shibata

**Affiliations:** Laboratory of Physiology and Pharmacology, School of Advanced Science and Engineering, Waseda University, Shinjuku-ku, Tokyo, Japan; Laboratory of Physiology and Pharmacology, School of Advanced Science and Engineering, Waseda University, Shinjuku-ku, Tokyo, Japan; Graduate School of Biomedical and Health Sciences, Hiroshima University, Hiroshima-shi, Hiroshima, Japan; Laboratory of Physiology and Pharmacology, School of Advanced Science and Engineering, Waseda University, Shinjuku-ku, Tokyo, Japan; Benesse Education Research and Development Institute, Benesse Corporation, Tama-shi, Tokyo, Japan; Benesse Education Research and Development Institute, Benesse Corporation, Tama-shi, Tokyo, Japan; Benesse Education Research and Development Institute, Benesse Corporation, Tama-shi, Tokyo, Japan; Benesse Education Research and Development Institute, Benesse Corporation, Tama-shi, Tokyo, Japan; Benesse Education Research and Development Institute, Benesse Corporation, Tama-shi, Tokyo, Japan; Institute for Liberal Arts, Tokyo Institute of Technology, Meguro-ku, Tokyo, Japan; Laboratory of Physiology and Pharmacology, School of Advanced Science and Engineering, Waseda University, Shinjuku-ku, Tokyo, Japan

**Keywords:** academic performance, Athens Insomnia Scale, boys, girls, Munich ChronoType Questionnaire, sex difference

## Abstract

**Study Objectives:**

Sleep problems, such as accumulated sleep loss and social jetlag (SJL), which is characterized by a discrepancy in a person’s sleep pattern between the weekday and the weekend, are associated with physical and mental health problems, and academic performance in young ages. However, sex differences in these associations are not fully understood. The purpose of this study was to investigate the effect of sex on sleep-related factors, mental health (negative mood), and academic performance in Japanese children and adolescents.

**Methods:**

A cross-sectional online survey was conducted with 9270 students (boys: *N* = 4635, girls: *N* = 4635) ranging from the fourth grade of elementary school to the third grade of high school, which typically includes ages 9–18 years in Japan. Participants completed the Munich ChronoType Questionnaire, the Athens Insomnia Scale, self-reported academic performance, and negative mood-related questions.

**Results:**

School grade-related changes in sleep behavior (e.g. delayed bedtime, shortened sleep duration, and increased SJL) were detected. Girls had greater sleep loss on weekdays and SJL on weekends than boys. Multiple regression analysis revealed that sleep loss and SJL were more associated with negative mood and higher insomnia scores in girls than in boys, but not with academic performance.

**Conclusions:**

Sleep loss and SJL in Japanese girls had a higher correlation to their negative mood and tendency to insomnia than in boys. These results suggest the importance of sex-dependent sleep maintenance for children and adolescents.

**Statement of Significance**

From a large-scale cross-sectional study, we identified significant sex differences in sleep behavior in Japanese children and adolescents (9–18 years old). Girls slept longer and woke up later on weekends than boys, suggesting that they might not have enough sleep on weekdays. The study also found that girls’ sleep loss had a greater impact on their negative mood than boys’ sleep loss. These results suggest the importance of sex-dependent sleep maintenance for children and adolescents.

## Introduction

Sleep deprivation (sleep loss) has been reported to be associated with an increase in various health risks, such as cardiovascular diseases, diabetes, metabolic syndrome, and depression, and has attracted attention as a global health issue [1–3]. A survey by the Organisation for Economic Co-operation and Development (OECD) found that Japanese people spend the lowest amount of sleep among 33 countries, revealing that Japan is one of the world’s most sleep-deprived countries (https://www.oecd.org/health/health-data.htm). In fact, according to a nationwide survey in Japan conducted in 2019, although the government recommends 6–8 hours of sleep, 37.5% of men and 40.6% of women sleep less than 6 hours a day [4].

A sleep loss-related sleep problem is “social jetlag” (SJL). SJL is caused by the “gap between social and biological time (circadian clock).” The circadian clock regulates many physiological functions with a day–night difference and modulates the sleep–wake cycle [5]. The SJL occurs when people wake up early for their social constraints (school and work) on weekdays, but their circadian clock phase is still delayed because of the delayed sleep phase on weekends [6, 7]. Previous studies have shown that SJL is associated with obesity and depression [8, 9]. Because evening chronotype people prefer to sleep later and to be active and alert in the evening, they tend to have more sleep loss on weekdays and more delayed bedtime and wake-up time on weekends with longer sleep, compared with morning chronotype. This sleep behavior in the evening chronotype people also results in a large SJL [7].

Adolescents tend to have more sleep deprivation than adults due to biological and social changes [10]. Biological changes include delayed circadian clock phase beginning around adolescence and delayed bedtimes due to delayed accumulation of sleep pressure [11, 12]. In addition, adolescents must cope with social changes, such as extracurricular activities, the use of electronic devices, and increased academic workloads, which can lead to sleep loss and other sleep disorders [13, 14]. One study showed that sleep deprivation in adolescents is a predictor of sleep disorders in adults [15], suggesting that adolescent sleep plays an important role in long-term health. Sleep duration in adolescents is decreasing year by year, with a delay in bedtime [16]. The National Sleep Foundation recommends 8–10 hours of sleep for 14- to 17-year-olds [17], but it has been reported that approximately 25% of adolescents in Japan sleep less than 6 hours, and sleep loss among adolescents is a growing problem in Japan [18]. SJL has also been reported in Japanese adolescents [19]. Sleep loss and SJL have been shown to be associated with a variety of health risks in both adults and adolescents and have negative effects on daily life, such as lower academic performance, negative mood, and daytime sleepiness [20–22]. However, most of these studies were conducted on high school and college students, and there have been few large-scale survey studies in younger ages such as children.

Sex differences in sleep have been observed in adolescents and adults [23, 24]. For example, circadian rhythms tend to be more delayed in men than in women between the ages of 20 and 40 [24]. On the other hand, subjective and objective data reported that girls generally tended to sleep longer than boys [23]. Women have been found to have a higher prevalence of sleep disorders, higher levels of daytime sleepiness, and longer desired sleep duration, suggesting that women may have a greater need for sleep than men [25, 26]. In addition, there are not only sex differences in sleep variables but also in the effects of sleep variables on health risk [27]. However, a large study of Japanese children and adolescents has not yet clarified the existence of sex differences in sleep variables or in associations with health risks.

In this study, we focused on the sex differences in sleep habits among Japanese elementary, junior high, and high school students, and how these sex differences affect academic performance, negative mood, and insomnia.

## Materials and Methods

### Ethical approval

The Ethics Review Committee on Research with Human Subjects at Waseda University approved this experiment (No. 2021-101) on June 4, 2021, and the guidelines of the Declaration of Helsinki were followed. This cross-sectional study was conducted, analyzed, and reported in accordance with the STROBE statement. Approval for data collection and use for research analysis was obtained from the participants when they answered the survey.

### Study design and participants

Using our previous cross-sectional web-based survey, we performed a power analysis for multiple regression analysis with confounding factors to detect the sample size [28]. An online survey company (Macromill Inc., Tokyo, Japan) was commissioned to conduct the current survey. The recruitment was done through the company’s online membership who registered a family structure. The company asked the online members who had children of targeted grades to participate, and then asked their children as participants to answer the questionnaire. We also asked parents to help their children to answer if necessary. Gift cards or shopping points were rewarded to the participants. Respondents who did not meet the criteria (e.g. mismatched grade or incomplete answer) were excluded by the company. Finally, 1030 participants were randomly selected from each grade level (from the fourth grade of elementary school (9–10 years old) to the third grade of high school [17–18 years old]), with a boys: girls ratio of 1:1. The survey was conducted in June 2021 and included 35 items related to basic characteristics (grade, sex, family structure), academic performance, mental health, and life habits (sleep, eating, and physical activity).

### Sleep loss and SJL

Sleep behaviors were calculated based on Munich ChronoType Questionnaire (MCTQ) [29]. This study focused on three sleep variables: (1) sleep loss across the week (SLOSSweek), which can be calculated by the difference in sleep duration of school days and free days. (2) SJL, which is calculated by the difference of midpoint of sleep on school days and free days. (3) Midpoint of sleep on free days corrected for sleep loss on workdays (MSFsc), which is an indicator of chronotype.

### Athens Insomnia Scale

Symptoms of insomnia were assessed using the Japanese version of the Athens Insomnia Scale (AIS)—Japanese version [30]. Each question of AIS was scored in the range of 0–3, and the total AIS score was in the range of 0–24. A high value of each item in the AIS indicates having more insomnia-related symptoms.

### Self-reported academic performance

Academic performance was assessed using self-evaluations of four subjects (Japanese, mathematics, science, and social studies) for elementary school students, and five subjects (Japanese, mathematics, science, social studies, and English) for junior and high school students. The questionnaire item was “What is your level of academic performance in your class or grade?” and this was done for each academic subject. Responses were rated on a five-point scale: 0, lower; 1, lower middle; 2, middle; 3, upper middle; and 4, upper. The total score of these responses was calculated and analyzed as a continuous variable, with a score of 0–16 for elementary school students and 0–20 for junior and high school students. Since the total number of subjects assessed differences between elementary, junior, and high school students, the analysis was conducted for each school type.

### Negative mood

The negative mood was assessed by five items: “fatigue,” “irritable mood,” “unmotivated,” “depressed,” and “poor appetite.” The responses were rated on a four-point scale: 0: “I don’t feel this at all,” 1: “I don’t feel this very much,” 2: “I feel this quite often,” and 3: “I feel this very much.” Therefore, the higher the number, the more negative the mood.

### Statistical analysis

Because most of the data did not pass the normality test, we chose nonparametric analysis in this study. To investigate the characteristics of the subjects by sex, Kruskal–Wallis tests were conducted for the sleep variable and the objective variables (academic performance, mood, and sleep) for grade changes, and Mann–Whitney *U*-tests were conducted for sex by grade.

Multiple regression analysis using the forced imputation method with an interaction term was then conducted to confirm the interaction effect of sex on the objective variables of SLOSSweek and SJL. Sex was treated as a dummy variable and placed as 0: “female” and 1: “male.” Grade (age difference) was scored as an ordinal variable and analyzed. We set grade as the control variable; sex as the interaction variable; SLOSSweek or SJL as the explanatory variable; and academic performance, mood, or sleep as the objective variable. Only those values for which an interaction effect was confirmed were subjected to a simple slope analysis, which was a subtest. In all multiple regression analyses, the coefficient of variance expansion was VIF <10 and there was no multicollinearity among the explanatory variables. All data were analyzed using the Statistical Package for the Social Sciences (SPSS ver. 27, IBM Corp., Chicago, IL), and a *p* value <0.05 indicated statistical significance. Data were expressed as the mean ± *SD*)or the mean ± *SEM*.

## Results

### Participants

In this study, data from 9270 students (4635 boys and 4635 girls) from the fourth year of elementary school through the third year of high school were used for analysis (Table 1). We confirmed significant differences in all sleep variables between elementary, junior, and high school students (*p* < 0.001 by Kruskal–Wallis test, Table 1).

**Table 1.** Basic characteristics.

		Sex	Elementary school	Junior high school	High school	Kruskal-Wallis test	Effect size ε2
Participants (N)		Boys	1545	1545	1545		
		Girls	1545	1545	1545		
BMI		Boys	18.07 ± 3.15	19.27 ± 3.02	20.31 ± 3.05	*p* < 0.001	0.096
		Girls	17.34 ± 3.25	18.80 ± 2.49	19.65 ± 2.95	*p* < 0.001	0.112
Wake-up time (hh:mm)	Weekdays	Boys	06:26 ± 00:32	06:29 ± 0:35	06:28 ± 00:39	*p* < 0.01	0.005
		Girls	06:27 ± 00:31	06:29 ± 00:33	06:20 ± 00:37	*p* < 0.001	0.019
	Free days	Boys	07:12 ± 01:05	07:53 ± 01:24	08:28 ± 01:37	*p* < 0.001	0.398
		Girls	07:36 ± 01:08	08:19 ± 01:24	08:31 ± 01:34	*p* < 0.001	0.402
Bedtime (hh:mm)	Weekdays	Boys	22:03 ± 00:44	22:56 ± 00:55	23:45 ± 00:59	*p* < 0.001	0.138
		Girls	22:03 ± 00:41	23:03 ± 00:57	23:44 ± 00:57	*p* < 0.001	0.089
	Free days	Boys	22:21 ± 00:52	23:16 ± 01:05	00:09 ± 01:09	*p* < 0.001	0.351
		Girls	22:23 ± 00:51	23:23 ± 01:03	00:04 ± 01:05	*p* < 0.001	0.338
Sleep duration (hh:mm)	Weekdays	Boys	08:23 ± 00:47	07:33 ± 00:52	06:45 ± 00:55	*p* < 0.001	0.386
		Girls	08:24 ± 00:44	07:26 ± 00:56	06:36 ± 00:59	*p* < 0.001	0.419
	Free days	Boys	08:23 ± 00:56	08:36 ± 01:11	08:18 ± 01:20	*p* < 0.001	0.045
		Girls	09:12 ± 00:59	08:56 ± 01:12	08:27 ± 01:22	*p* < 0.001	0.077
MSFsc (hh:mm)		Boys	02:33 ± 00:44	03:10 ± 00:55	03:43 ± 01:00	*p* < 0.001	0.24
		Girls	02:41 ± 00:44	03:18 ± 00:52	03:37 ± 00:55	*p* < 0.001	0.193
SJL (hh:mm)		Boys	00:35 ± 00:33	00:54 ± 00:43	01:12 ± 00:51	*p* < 0.001	0.127
		Girls	00:46 ± 00:36	01:05 ± 00:44	01:16 ± 00:49	*p* < 0.001	0.091
SLOSSweek (hh:mm)		Boys	01:02 ± 01:03	01:42 ± 01:31	02:22 ± 01:37	*p* < 0.001	0.134
		Girls	01:20 ± 01:10	02:14 ± 01:39	02:44 ± 01:57	*p* < 0.001	0.128

Data are expressed with mean ± standard deviations (SD).

### Grade- and sex-dependent sleep behavior

**Figure 1.**
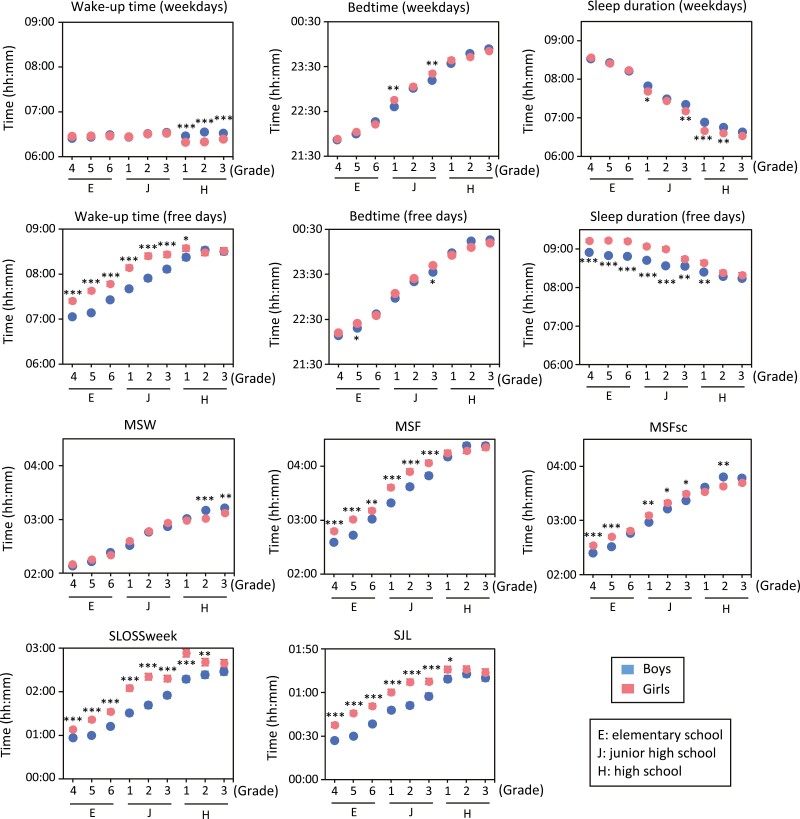
Sex differences in sleep variables in each grade. *p < 0.05; **p < 0.01; ***p < 0.001, between boys and girls by Mann–Whitney *U*-test. Data were expressed as mean ± *SEM*. “Grade” was started from 4 as the fourth grade of elementary school to 12 as the third grade of high school. MSW: midpoint of sleep on weekdays, MSF: midpoint of sleep on free days.

The sex differences in sleep variables according to grade are shown in Figure 1. On weekdays, there was a tendency for bedtime to become later and sleep duration to become shorter as students grade level progressed. Among high school students, girls showed significantly earlier wake-up times than boys, and had shorter sleep durations. On free days, both wake-up time and bedtime became later as the grade level increased, and sleep duration decreased. Girls woke up significantly later and slept longer than boys in most grades. It was also found that the midpoint of sleep time became later as the grade increased for both weekdays and free days, but a sex difference was only seen for free days in each grade. The chronotype (MSFsc) was significantly later in girls through junior high school, but the trend was the opposite in high school students. Importantly, both SLOSSweek and SJL tended to increase with grade and were significantly greater in girls than in boys in almost all grades.

### Sex differences in self-reported academic performance, negative mood, and insomnia

**Figure 2.**
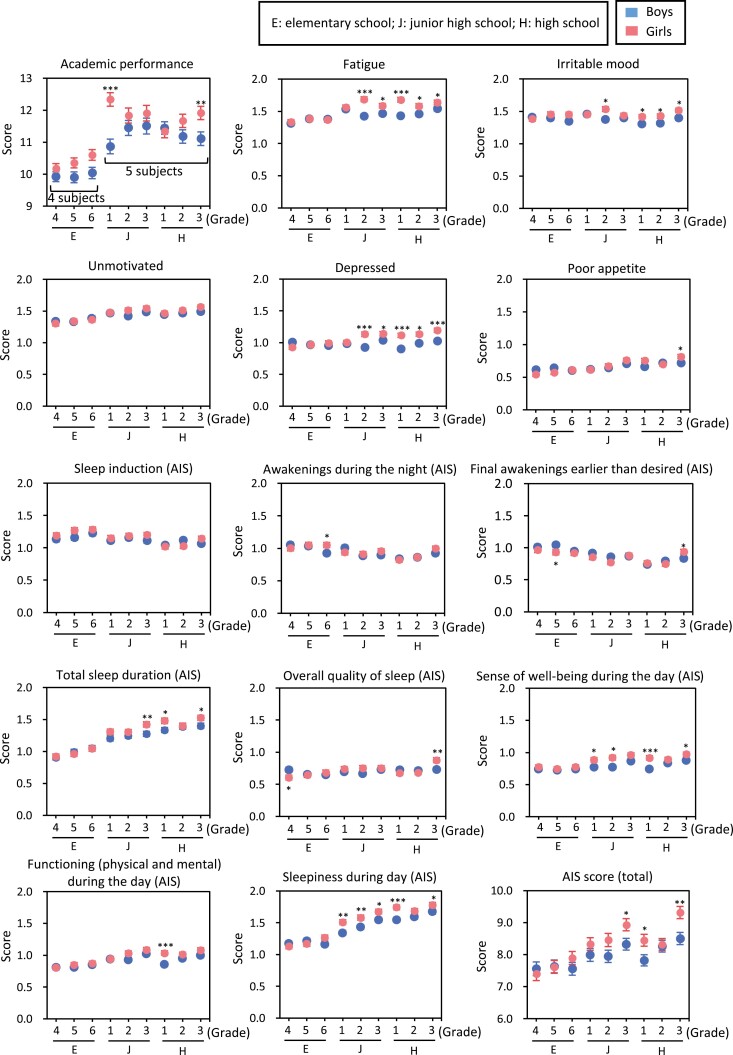
Sex differences in academic performance, mood, and sleep-related contents in the AIS in each grade. *p < 0.05; **p < 0.01; ***p < 0.001, between sex by Mann–Whitney *U*-test. Data were expressed as mean ± *SEM*. Higher score means higher academic performance or better mood. Each question of AIS was scored in the range of 0–3, and the total AIS score was in the range of 0–24. A higher value of each item in the AIS indicates having more insomnia-related problems. “Grade” was started from 4 as the fourth grade of elementary school to 12 as the third grade of high school.

Sex differences in self-reported academic performance, negative mood, and insomnia scale by grade are shown in Figure 2. In some grade groups, academic performance was higher in girls than in boys. Girls scored higher in negative moods than did boys. In the content of the AIS questionnaire, a sex difference was found in many grades for the items “sense of well-being during the day” and “sleepiness during the day.” However, the quality of sleep showed no consistent difference between sex. The total AIS score was also higher in girls than in boys in some grade groups.

### Interaction effects of sex on the association between SLOSSweek/SJL with other parameters

**Figure 3.**
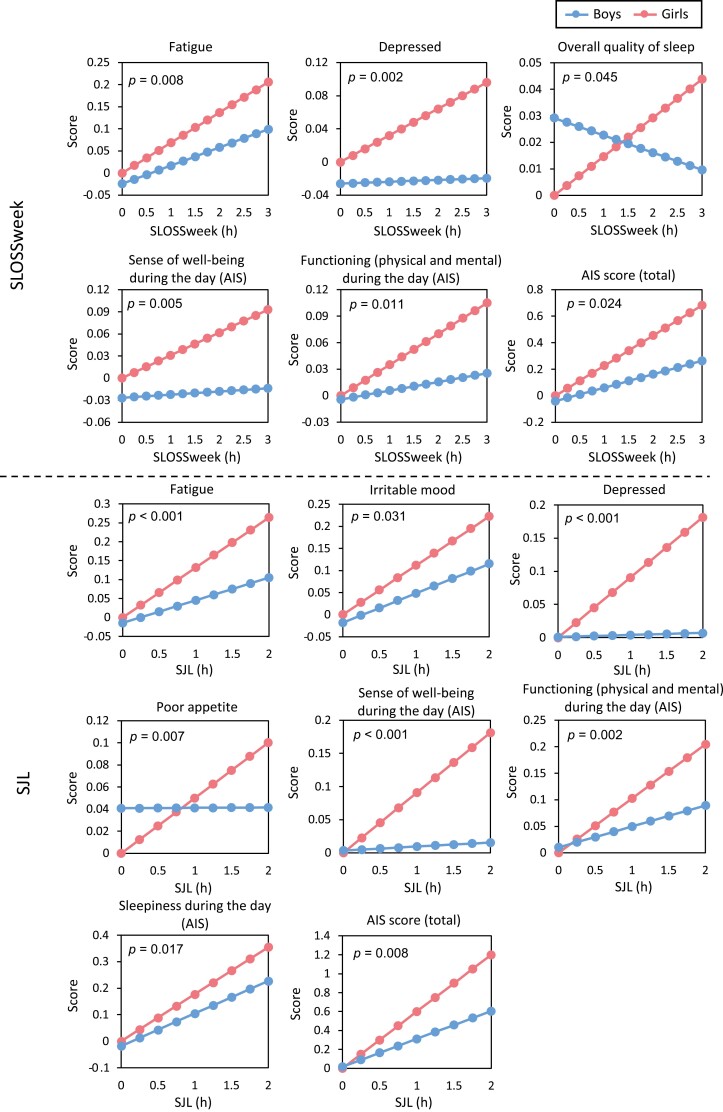
Interaction plot: effects of sex between SLOSSweek/SJL, and academic performance, mood, and sleep. Girls with 0 hour of SLOSSweek/SJL were set as 0 in each graph. *p* value in each graph refers to the interaction term between SLOSSweek/SJL and sex (SLOSSweek × sex or SJL × sex) in the multiple regression analysis in Table 2.

Multiple regression analyses were conducted to investigate the interaction effect of sex on the association of SLOSSweek/SJL with self-reported academic performance, negative mood, or insomnia. The results of the multiple regression analysis are shown in Table 2, and those of the simple slope analysis are shown in Table 3. In addition, simple slopes for each sex are shown in the graphs (Figure 3). These were graphed by placing the estimate for girls (*z* = 0) and SLOSSweek or SJL = 0 at 0. All variables of self-reported academic performance, negative mood, and AIS contents were significantly associated with SLOSSweek and SJL (Table 2). In terms of academic performance, no interaction effect of SLOSSweek and sex was identified in all types of elementary, junior, and high school (Table 2). On the other hand, a significant interaction effect was found for “fatigue” and “depressed.” For “fatigue,” there was a significant positive slope for both sexes, and the slope was larger for girls than for boys (Table 3 and Figure 3). In addition, only girls showed a significant positive slope for “depressed,” indicating that the level of SLOSSweek in girls has a greater impact on their mood than in boys (Figure 3). A significant negative interaction effect was found for the following sleep categories: “overall quality of sleep,” “sense of well-being during the day,” “functioning (physical and mental) during the day,” and “AIS score.” “AIS score” showed a significant positive slope for both sexes, with a larger slope for girls. For the other items, a significant positive slope was found only for the girls. Therefore, SLOSSweek in girls has a more negative impact on overall quality of sleep, sense of well-being during the day, functioning (physical and mental) during the day, and AIS score, than in boys.

**Table 2.** Effects of sex between sleep variables (SLOSSweek or SJL) and academic performance, mood, and sleep with multiple regression analysis

		SLOSSweek				SJL			
		*B*	*SE*	*p*	*R*-Squared	*B*	*SE*	*p*	*R*-Squared
(A)Academic performance									
Sleep variables (SLOSSweek or SJL)	Elementary school	−0.34	0.08	<0.001	0.011	−0.62	0.15	<0.001	0.01
	Junior high school	−0.27	0.08	<0.001	0.011	−0.91	0.18	<0.001	0.015
	High school	−0.27	0.06	<0.001	0.011	−0.77	0.14	<0.001	0.014
Sex	Elementary school	−0.69	0.20	<0.001		−0.71	0.19	<0.001	
	Junior high school	−0.92	0.30	0.003		−1.32	0.31	<0.001	
	High school	−0.66	0.28	0.020		−0.86	0.29	0.003	
Sleep variables × Sex	Elementary school	0.16	0.12	0.174		0.30	0.21	0.165	
	Junior high school	0.01	0.12	0.927		0.42	0.25	0.092	
	High school	0.07	0.09	0.424		0.35	0.19	0.071	
(B) Mood									
Sleep variables (SLOSSweek or SJL)	Fatigue	0.069	0.007	<0.001	0.024	0.132	0.016	<0.001	0.02
	Irritable mood	0.049	0.007	<0.001	0.01	0.111	0.015	<0.001	0.01
	Unmotivated	0.064	0.007	<0.001	0.02	0.160	0.015	<0.001	0.025
	Depressed	0.032	0.007	<0.001	0.009	0.091	0.015	<0.001	0.011
	Poor appetite	0.018	0.006	0.003	0.008	0.050	0.013	<0.001	0.009
Sex	Fatigue	−0.024	0.026	0.354		−0.014	0.027	0.594	
	Irritable mood	−0.037	0.025	0.143		−0.018	0.026	0.480	
	Unmotivated	0.015	0.025	0.555		0.030	0.026	0.244	
	Depressed	−0.026	0.024	0.280		0.001	0.025	0.966	
	Poor appetite	0.016	0.022	0.468		0.041	0.023	0.071	
Sleep variables × Sex	Fatigue	−0.027	0.010	0.008		−0.072	0.022	<0.001	
	Irritable mood	−0.011	0.010	0.265		−0.045	0.021	0.031	
	Unmotivated	−0.010	0.010	0.330		−0.036	0.021	0.088	
	Depressed	−0.030	0.010	0.002		−0.088	0.020	<0.001	
	Poor appetite	−0.011	0.009	0.204		−0.050	0.018	0.007	
(C) Sleep (AIS)									
Sleep variables (SLOSSweek or SJL)	Sleep induction	0.012	0.008	0.123	0.005	0.082	0.017	<0.001	0.01
	Awakenings during the night	−0.007	0.008	0.330	0.004	0.019	0.017	<0.001	0.004
	Final awakenings earlier than desired	−0.030	0.008	<0.001	0.01	−0.065	0.017	<0.001	0.01
	Total sleep duration	0.091	0.008	<0.001	0.058	0.161	0.018	<0.001	0.053
	Overall quality of sleep	0.015	0.007	0.047	0.002	0.033	0.016	0.042	0.002
	Sense of well-being during the day	0.031	0.007	<0.001	0.01	0.091	0.015	<0.001	0.012
	Functioning (physical and mental) during the day	0.035	0.007	<0.001	0.013	0.102	0.015	<0.001	0.015
	Sleepiness during day	0.081	0.008	<0.001	0.069	0.177	0.017	<0.001	0.069
	AIS score	0.227	0.039	<0.001	0.013	0.600	0.085	<0.001	0.015
Sex	Sleep induction	−0.061	0.028	0.031		−0.044	0.029	0.131	
	Awakenings during the night	0.004	0.028	0.894		0.009	0.028	0.740	
	Final awakenings earlier than desired	0.049	0.028	0.073		0.041	0.028	0.149	
	Total sleep duration	0.003	0.029	0.911		−0.009	0.030	0.766	
	Overall quality of sleep	0.029	0.027	0.272		0.025	0.027	0.354	
	Sense of well-being during the day	−0.027	0.024	0.268		0.003	0.025	0.891	
	Functioning (physical and mental) during the day	−0.004	0.025	0.865		0.010	0.025	0.692	
	Sleepiness during day	−0.034	0.028	0.220		−0.019	0.028	0.511	
	AIS score	−0.040	0.141	0.774		0.018	0.143	0.903	
Sleep variables × Sex	Sleep induction	0.017	0.011	0.141		0.023	0.023	0.324	
	Awakenings during the night	−0.015	0.011	0.175		−0.029	0.023	0.203	
	Final awakenings earlier than desired	−0.021	0.011	0.061		−0.029	0.023	0.194	
	Total sleep duration	−0.017	0.012	0.132		−0.031	0.024	0.194	
	Overall quality of sleep	−0.021	0.011	0.045		−0.038	0.022	0.085	
	Sense of well-being during the day	−0.027	0.010	0.005		−0.085	0.020	<0.001	
	Functioning (physical and mental) during the day	−0.025	0.010	0.011		−0.063	0.020	0.002	
	Sleepiness during day	−0.017	0.011	0.125		−0.054	0.023	0.017	
	AIS score	−0.126	0.056	0.024		−0.306	0.116	0.008	

Multivariable regression analyses adjusted for grade (age). *B*: partial regression coefficient, Sleep × Sex: interaction term between sleep variables (SLOSSweek or SJL) and sex.

**Table 3.** Results of simple slope analysis

	Sex	SLOSSweek			SJL		
		*B*	*SE*	*p*	*B*	*SE*	*p*
(B) Mood							
Fatigue	Boys	0.041	0.008	<0.001	0.060	0.015	<0.001
	Girls	0.069	0.007	<0.001	0.132	0.016	<0.001
Irritable mood	Boys	—	—	—	0.067	0.015	<0.001
	Girls	—	—	—	0.111	0.015	<0.001
Unmotivated	Boys	—	—	—	—	—	—
	Girls	—	—	—	—	—	—
Depressed	Boys	0.002	0.007	0.768	0.003	0.014	0.834
	Girls	0.032	0.007	<0.001	0.091	0.015	<0.001
Poor appetite	Boys	—	—	—	0.000	0.013	0.978
	Girls	—	—	—	0.050	0.013	<0.001
(C) Sleep (AIS)							
Sleep induction	Boys	—	—	—	—	—	—
	Girls	—	—	—	—	—	—
Awakenings during the night	Boys	—	—	—	—	—	—
	Girls	—	—	—	—	—	—
Final awakenings earlier than desired	Boys	—	—	—	—	—	—
	Girls	—	—	—	—	—	—
Total sleep duration	Boys	—	—	—	—	—	—
	Girls	—	—	—	—	—	—
Overall quality of sleep	Boys	−0.006	0.008	0.417	—	—	—
	Girls	0.015	0.007	0.047	—	—	—
Sense of well-being during the day	Boys	0.004	0.007	0.552	0.006	0.014	0.663
	Girls	0.031	0.007	<0.001	0.091	0.015	<0.001
Functioning (physical and mental) during the day	Boys	0.01	0.008	0.190	0.04	0.015	0.007
	Girls	0.035	0.007	<0.001	0.102	0.015	<0.001
Sleepiness during day	Boys	—	—	—	0.123	0.016	<0.001
	Girls	—	—	—	0.177	0.017	<0.001
AIS score	Boys	0.102	0.042	0.017	0.294	0.083	<0.001
	Girls	0.227	0.039	<0.001	0.600	0.085	<0.001

Multivariable regression analyses adjusted for grade (age). *B*: partial regression coefficient. (A) Academic performance is omitted because the interaction effect could not be confirmed.

In academic performance, similar to the SLOSSweek, no interaction effect of SJL and sex was found at all school types (Table 2). In terms of mood, four items showed significant negative interaction effects: “fatigue,” “irritable,” “depressed,” and “poor appetite” (Table 3). In addition, the items “depressed” and “poor appetite” were found to have a significant positive slope only for girls (Figure 3). In the case of sleep, a significant negative interaction effect was found for the five items of “sense of well-being during the day,” “functioning (physical and mental) during the day,” “sleepiness during day,” and “AIS score.” For “sense of well-being during the day,” only girls showed a significant positive slope. For the other items, there was a significant positive slope for both sexes, and the slope was larger for girls. Thus, SJL has a stronger negative impact on “sense of well-being during the day,” “functioning (physical and mental) during the day,” “sleepiness during the day,” and “AIS scores” in girls than in boys.

## Discussion

In this study, we found that girls experienced greater sleep loss and SJL. Girls slept longer and woke up later on free days than boys in almost all grades (9–18 years old), suggesting that girls in Japan did not have enough sleep and have a larger sleep debt than boys. These sex differences in sleep characteristics were consistent with the recent national survey database “Survey on Time Use and Leisure Activities (2016)” by the Statistics Bureau of Japan [31]. However, no one has focused on this sex difference in Japanese children and adolescents.

Several studies in other countries have demonstrated longer sleep duration in school-aged girls than boys, especially on free days [32, 33], which is consistent with our results. However, one study showed no sex difference in adolescents’ sleep behavior, which might be due to the small sample size [34]. In addition, our SLOSSweek and SJL calculation based on the MCTQ questionnaire identified greater sleep loss and SJL in girls than in boys. The evening chronotype is associated with a larger SJL [7], and the larger SJL in girls identified in the current study might be due to greater sleep loss on weekdays. This is because the sex difference was only detected at wake-up time, but not at bedtime on free days. It seems that sleep loss-induced longer sleep occurred on free days in girls, which induced later MSF and larger SJL. The mechanism of this sex difference in sleep characteristics might be explained by several factors, including physiological and social aspects. Generally, women have been found to have a higher sleep needs than men, because women have a higher prevalence of sleep disorders, higher levels of daytime sleepiness, and longer desired sleep duration [23, 24]. In addition, since we did not assess the use of an alarm clock in this survey, it remains unclear whether the observed earlier awakenings in females are natural occurrences or due to morning obligations.

However, a recent study in young mice (8–12 weeks old) showed the opposite results, in which female mice showed shorter sleep duration and smaller response to sleep deprivation than male mice [35]. Thus, further investigation is needed to determine sex differences in sleep physiology in the future.

Girls in high school woke up significantly earlier than boys on weekdays, which may be because girls require more time for dressing and grooming. A survey on time use and leisure activities (2016) has shown that the average time required to get ready for school becomes longer for girls than boys as they advance through school types [31]. Taken together, sleep loss occurs among adolescents worldwide, and seems to be of particular health concern for females.

In the current study, we found that sleep loss and SJL in girls were more correlated with negative mood and insomnia-related problems, but not with self-perceived academic performance, than in boys. Girls had higher academic performance than boys, as reported previously [36, 37]. However, previous studies have not yielded consistent results on the effect of sex on sleep and academic performance [38–40], suggesting that the effect might be small or that the girls are studying even if they lose sleep. As shown in Figure 2, girls showed higher irritable/depressed mood, fatigue, and daytime sleepiness than boys, which was consistent with previous studies [41, 42]. Regarding the moderating effect of sex on mood and sleep in the current study, five previous studies reached a conclusion similar to the current results. Agathão et al. demonstrated that short sleep duration seemed to be problematic for common mental disorders in girls [42]. Mathew et al. reported that the Center for Epidemiologic Studies Depression Scale (CES-D) was correlated with shorter sleep time and larger SJL in female adolescents than in boys, but there was no mean difference in SJL between the sexes [43]. Conklin reported that chronic shorter sleep (<8 hours) on weekdays was more associated with depressed mood in girls than in boys (age 13–18 years) [44]. An intervention study conducted among 14- to 18-year-olds in Australia found that girls showed greater sensitivity to the effects of sleep deprivation on various moods, including anger, anxiety, and fatigue [45]. In addition, a study conducted in South Korea in 2017 reported that the effect of short sleep duration on suicidal ideation among girls was 2.5 times higher than that of boys, from the first year of junior high school to the third year of high school [46].

Previous studies in adults have shown that the cortisol arousal response (CAR), a biomarker of stress response, is greater with shorter sleep durations [47–49]. In addition, CAR has been reported to increase in women, but to decrease in men, with greater depressive symptoms, which may account for women’s vulnerability to sleep deprivation and mood [50]. However, studies on sleep deprivation and CAR in adolescents are scarce, and consistent results have not been obtained, suggesting that more detailed studies in adolescents are needed [51]. SJL is a measure of the misalignment between endogenous and exogenous rhythms [7]. Previous studies have shown that disruptions in circadian rhythms have adverse effects on endocrine functions, such as decreased 24-hour melatonin levels and altered cortisol secretion patterns, which have been reported to contribute to the development of depressive symptoms [52, 53]. SJL and CAR have also been shown to be correlated [54]. Thus, there are combined effects of sleep loss and SJL on mood, and future studies should address both issues carefully.

### Limitations

The limitations of our study include misclassification due to self-reporting by the adolescents, unmeasured and uncontrolled confounding factors (e.g. residence area, family income, or family composition). We did not ask whether participants use an alarm clock on free days in this MCTQ questionnaire, which might be a limitation of the current analysis. Additionally, our mood-questionnaire was not specifically designed for children and adolescents. A more objective methodology, including actigraphy recordings and observation methods such as sleep diaries, is desired. The cross-sectional study design limits the determination of the causal links among all variables.

## Conclusion

In this study, we observed higher rates of sleep loss and SJL in girls than in boys, and the moderating effect of sex on the association of sleep loss and SJL on negative mood among Japanese elementary, middle, and high school students. The results of this study suggest the importance of sex-dependent sleep care for children and adolescents.

## Data Availability

The data used in this study are the property of the company and will not be released to the public. However, the data will only be provided to researchers upon request for research purposes.
